# Modulatory Effects of Estradiol and Its Mixtures with Ligands of GPER and PPAR on MAPK and PI3K/Akt Signaling Pathways and Tumorigenic Factors in Mouse Testis Explants and Mouse Tumor Leydig Cells

**DOI:** 10.3390/biomedicines10061390

**Published:** 2022-06-12

**Authors:** Ewelina Gorowska-Wojtowicz, Michal Duliban, Malgorzata Kotula-Balak, Barbara Bilinska

**Affiliations:** 1 Department of Medical Physiology, Jagiellonian University Medical College, Michalowskiego 12, 31-126 Krakow, Poland; 2 Department of Endocrinology, Institute of Zoology and Biomedical Research, Jagiellonian University in Krakow, Gronostajowa 9, 30-387 Krakow, Poland; michal.duliban@doctoral.uj.edu.pl; 3 University Centre of Veterinary Medicine, University of Agriculture in Krakow, Mickiewicza 24/28, 30-059 Krakow, Poland; malgorzata.kotula-balak@urk.edu.pl

**Keywords:** testis, Leydig cells, estrogens, G protein-coupled estrogen receptor (GPER), peroxisome proliferator-activated receptors (PPARs), GPER agonist/antagonist, PPAR antagonists

## Abstract

The present study was designed to evaluate how estradiol alone or in combination with G protein-coupled estrogen receptor (GPER) agonists and GPER and peroxisome proliferator-activated receptor (PPAR) antagonists alter the expression of tumor growth factor β (TGF-β), cyclooxygenase-2 (COX-2), hypoxia inducible factor 1-alpha (HIF-1α), and vascular endothelial growth factor (VEGF) in mouse testis explants and MA-10 mouse tumor Leydig cells. In order to define the hormone-associated signaling pathway, the expression of MAPK and PI3K/Akt was also examined. Tissue explants and cells were treated with estradiol as well as GPER agonist (ICI 182,780), GPER antagonist (G-15), PPARα antagonist (GW6471), and PPARγ antagonist (T00709072) in various combinations. First, we showed that in testis explants GPER and PPARα expressions were activated by the GPER agonist and estradiol (either alone or in mixtures), whereas PPARγ expression was activated only by GPER agonist. Second, increased TGF-β expression and decreased COX-2 expression were found in all experimental groups of testicular explants and MA-10 cells, except for up-regulated COX-2 expression in estradiol-treated cells, compared to respective controls. Third, estradiol treatment led to elevated expression of HIF-1α and VEGF, while their lower levels versus control were noted in the remaining groups of explants. Finally, we demonstrated the up-regulation of MAPK and PI3Kp85/Akt expressions in estradiol-treated groups of both ex vivo and in vitro models, whereas estradiol in mixtures with compounds of agonistic or antagonistic properties either up-regulated or down-regulated signaling kinase expression levels. Our results suggest that a balanced estrogen level and its action together with proper GPER and PPAR signaling play a key role in the maintenance of testis homeostasis. Moreover, changes in TGF-β and COX-2 expressions (that disrupted estrogen pathway) as well as disturbed GPER-PPAR signaling observed after estradiol treatment may be involved in testicular tumorigenesis.

## 1. Introduction

Estrogens play an important role in the regulation of normal physiological conditions and pathological processes in many disease states [1]. In the male, numerous studies show that the primary target for estradiol (a main estrogen produced in testes) are Leydig cells equipped with estrogen receptors α and β (ERα and ERβ) that mediate the hormone action in the classical manner [2,3,4]. In the testis, the expression of ERs varies mainly according to the developmental stage, cell type and species [5,6]. Estradiol also modulates spermatogenesis in a cell-specific manner, germ cell proliferation, differentiation, as well as germ cell survival and apoptosis [7]. Despite multiple studies, the mechanism of estradiol’s action and the role of estrogen receptors in testis are still debatable, as estradiol mediates not only classical signaling but also rapid signaling via pathways that involve a membrane estrogen receptor—the G-protein-coupled estrogen receptor (GPER; formerly known as GPR30) [8,9,10]. This receptor was cloned for the first time by the research groups in the 1990s [11,12]. It is a seven-transmembrane-domain receptor functioning autonomously from classical ERs and ubiquitously expressed in many organs including the brain, heart, lung, pancreas, and male and female reproductive organs [13,14,15]. Among others, the expression of GPER has been detected in rat and human myocardium and cultured cardiomyocytes [16,17], in adult female rat hippocampus [18], in mouse kidney [19], in human and mouse somatic cells of the male gonad [20,21], in human and pig spermatozoa [22], and sheep testis and epididymis [23].

Evidence over the last 15 years has revealed that GPER regulates transcriptional activity and mediates not only genomic but also rapid estradiol responses via the activation of kinases, MAPK/ERK and PI3K, generation of cAMP, mobilization of intracellular calcium stores, and activation of metalloproteinases [24,25]. Based on the observation that GPER is involved in the stimulation of ERK1/2 phosphorylation it was suggested that GPER may also act as an aldosterone receptor [26]. Recently, De Francesco and co-workers demonstrated modulation of GPER expression by vascular endothelial growth factor (VEGF) involved in angiogenesis in healthy and tumor tissues [27]. Currently, multiple GPER knock-out models help to elucidate a role for this receptor in physiology and pathophysiology [28].

It should be emphasized that our previous study revealed cross-talk between GPER and peroxisome proliferator-activated receptors, PPARα and PPARγ, leading to reduced expression of PPARα and increased expression of PPARγ in mouse Leydig cells after the GPER blockage. This interaction regulates the steroidogenic function of Leydig cells throughout life, controlling their morphology and behaviour [29]. Moreover, in a further study on mouse testes with experimentally induced human Leydig cell tumor (LCT), we demonstrated VEGF overexpression and hypoxia inducible factor 1 subunit α (HIF-1α) downregulation with a concomitant decrease of GPER activity [30]. Altogether, we detected both GPER and PPAR expressions in mouse Leydig cells in vivo and in vitro, in human Leydig cells, and in LCTs [29,30].

It is worth adding that PPARs belong to the superfamily of nuclear steroid receptors and are widely distributed in cells. After ligand binding, PPARs act as transcription factors [31]. Aberrant activation of PPAR signaling may be involved (after disturbance of lipid metabolism) in abnormalities of hormonal balance and functioning of the male and female reproductive systems [29,30,32]. Moreover, both GPER and PPARs can bind sex steroids and hormone-like chemicals [33]. As shown by Kotula-Balak et al. [34], estradiol secretion by MA-10 cells is altered after GPER, PPARα and PPARγ blockage by GPER and PPARs antagonists (G-15, GW6471, and T00709072), respectively. The hormone concentration markedly increased after treatment with G-15 and G-15+GW6471, significantly decreased in cells treated with T00709072 and G-15+T00709072, and was unchanged after GW6471 alone. The authors suggested the GPER/PPARα-mediated control of estradiol secretion. In addition, we demonstrated that the GPER/PPARα-mediated control is realized either via PI3K-Akt-mTOR pathway in MA-10 cells or only via mTOR pathway in human LCT [34]. Moreover, Lee et al. [35] reported that ligand activation of PPARγ modulates transforming growth factor (TGF-β1)-mediated gene regulation. TGF-β is a pleiotropic cytokine with the potent regulatory and inflammatory activity that controls proliferation and differentiation in most cell types [36,37] and can inhibit growth processes by blocking the cell cycle in the middle and late G1 phase [38]. Furthermore, in various pathological processes such as cancer and inflammation, cyclooxygenase-2 (COX-2) is also implicated, and its involvement in the development and progression of breast cancer is well recognized [39,40]. It should be stressed, however that COX-2 is implicated by its overexpression in carcinogenesis not only in rat testis and human breast cancer, but also in most solid human cancers such as prostate, lung, colorectal, pancreatic, liver [41] and in transgenic mouse models [42]. Moreover, COX-2 plays a role in inflammation processes, e.g., in rat kidneys [43] or human gastric tumors [44]. In the latter study, COX-2 and its downstream product, prostaglandin E2, have been reported as a key player in generation of the inflammatory microenvironment [44].

In light of the above findings, we decided to evaluate whether and how the estradiol alone or in combinations with GPER agonist and GPER and PPARs antagonists affect the protein expression of tumorigenic and angiogenic factors, TGF-β, COX-2, HIF-1α, and VEGF, in both ex vivo and in vitro models such as mouse testis explants and mouse tumor Leydig cells. Moreover, the expression levels of PI3K/Akt and MAPK signaling proteins were examined to define the estradiol-mediated pathways in the explants and Leydig cells. This study follows our previously reported data [29] and provides further knowledge on the biology of Leydig cells in normal and pathological conditions.

It should be added that in our earlier study we measured testosterone and estradiol concentrations in mouse testes and progesterone secretion by MA-10 cells in vitro. After treatment with GPER antagonists, intratesticular testosterone concentration significantly increased in immature mice and decreased in mature individuals, while intratesticular estradiol concentrations were significantly lower in immature and aged males compared to those in mature animals, indicating age- and GPER-dependent steroid hormone production in Leydig cells [21].

Finally, it is noteworthy that an ex vivo model is ethically advantageous, requires less number of animals (many explants may be isolated from one individual), provides tightly controlled conditions, and reduces the effects of variability between animals (control and treated testis explants may be isolated from one individual) in comparison with an in vivo experiment. Moreover, such a model allows to investigate the direct effects of different chemicals on testicular tissue regulatory mechanisms which remain largely unknown.

## 2. Materials and Methods

### 2.1. Animals and Testis Explants’ Culture Conditions

Sexually mature male mice (C57BL/6; *n* = 10) were obtained from the Department of Genetics and Evolution, Institute of Zoology and Biomedical Research, Jagiellonian University in Krakow, Poland. Animals were maintained in 12 h dark-light cycle (250 lux at cages level) in room temperature (22 °C) and 55% relative humidity with ad libitum feeding and water until three months of age when they were sacrificed, after which the testes were immediately removed, trimmed free of access fat and connective tissue and cut into small pieces (approx. 2 mm). Sacrificing mice for testis collection was performed in accordance with Polish regulations (Act of 15 January 2015 on the protection of animals used for scientific and educational purposes; Journal of Laws of 2015, item 266).

Mouse testis explants were cultured in M199 medium (Gibco, Grand Island, NY, USA) supplemented with 10% normal horse serum (NHS) and the antibiotic mixture (100 IU/mL penicillin and 100 µg/mL streptomycin) (Gibco) on the surface of a 1.5% agar clot (Bio-Rad Laboratories, GmbH, Munich, Germany). Tissues were cultured in 6-well dishes and maintained at 37 °C in a humidified atmosphere of 95% air and 5% CO_2_ for three days. Twenty-four hours before the experiments, the medium was removed and replaced with a medium without phenol red supplemented with 5% dextran coated, charcoal-treated NHS (5% DC-NHS) to exclude estrogenic effects caused by the medium.

### 2.2. Mouse Leydig Cell Line (MA-10) Culture Conditions

Mouse tumor Leydig cell line (MA-10) was obtained from American Type Culture Collection (ATCC, Manassas, VA, USA). The cells were cultured in 75 cm^2^ bottles coated with 0.1% gelatin (ATCC) in DMEM:F12 medium (containing 2.5 mM L-glutamine, 15 mM HEPES, 0.5 mM sodium pyruvate, and 1200 mg/L sodium bicarbonate) supplemented with 15% normal horse serum (NHS, PAN-Biotech GmbH; Aidenbach, Germany), 20 mM HEPES (Sigma-Aldrich; St. Louis, MO, USA), and 1% penicillin/streptomycin mixture (Gibco, Grand Island, NY, USA). Semi-confluent cell cultures, initially seeded in 6-well culture dishes at a density of 1 × 10^7^ cells per well for lysate preparation, were maintained at 37 °C in a humidified atmosphere of 5% CO_2_ for two to three days with everyday replacement of the medium for the fresh one. Twenty-four hours before the experiments, the medium was removed and replaced with a medium without phenol red supplemented with 5% dextran-coated, charcoal-treated FBS (5% DC-FBS) to exclude estrogenic effects caused by the medium as described previously [29].

### 2.3. Testis Explants and MA-10 Cells Treatments

Both the tissue explants and MA-10 cells were allotted into control and experimental groups treated for 24 h with selected chemicals dissolved in dimethyl sulfoxide (DMSO) (Sigma-Aldrich). Control explants and control MA-10 cells were incubated with a vehicle (0.1% DMSO) only. For experiments, the explants and MA-10 cells were treated with GPER agonist (ICI 182,780; 50 nM; Sigma-Aldrich), GPER antagonist (G-15; (3aS*,4R*,9bR*)-4-(6-bromo-1,3-benzodioxol-5yl)-3a,4,5,9b-3H-cyclopenta[c]quinolone; 10 nM; Tocris Bioscience, Bristol, UK), PPARα antagonist (GW647; N-((2S)-2-(((1Z)-1-methyl-3-oxo-3-(4-(trifluoromethyl)phenyl)prop-1-enyl)amino)-3-(4-(2-(5-methyl-2-pheny1-1,3-oxazol-4-yl)ethoxy)phenyl)propyl)propanamide; 10 nM; Tocris Bioscience), PPARγ antagonist (T00709072, 2-chloro-5-nitro-N-4-pyridinylbenzamide; 10 nM; Tocris Bioscience), and estradiol (E2; 100 nM; Sigma-Aldrich) either alone or in combination with each of the compounds (100 nM E2+50 nM ICI 182,780, 100 nM E2+10 nM G-15, 100 nM E2+10 nM GW6471, and 100 nM E2+10 nM T00709072).

The compounds used for explant treatment and MA-10 cell incubations were freshly prepared as 100 nM stock solutions and stored at −20 °C. Stock concentrations were subsequently dissolved in M199 medium to final concentrations. Chosen doses were based on literature data [45,46,47] and our own [29,30]. In the preliminary studies, dose range (1, 10, 50, 100 μM and 1, 10, 50, 100 nM) was tested and doses of 50 nM ICI 182,780; 10 nM G-15; 10 nM GW6471; 10 nM T00709072, and 100 nM (E2) were selected experimentally as high enough to produce changes in the expression of the proteins studied. Both the explant homogenates and cell lysates were frozen at −80 °C and used for gene expression analysis at protein levels.

### 2.4. Western Blot Analysis

Homogenates and lysates were obtained by sample sonication with a cold Tris/EDTA buffer (50 nM Tris, 1 mM EDTA, 7.5 pH), supplemented with broad-spectrum protease inhibitors (Sigma-Aldrich). The protein concentrations were measured by the Bio-Rad DC Protein Assay Kit with BSA as a standard (Bio-Rad Laboratories, Hercules, CA, USA). Equal amounts of protein were denatured and separated using SDS-PAGE electrophoresis and transferred to a PVDF membrane (Merck Millipore, Darmstadt, Germany) and analyzed by western blotting with the respective primary antibodies (see Table 1).

The presence of the primary antibody was revealed with horseradish peroxidase-conjugated secondary antibodies diluted 1:3000 (Vector Lab., Burlingame, CA, USA). Immunoreactive proteins were detected using chemiluminescence with luminol reagent (Sigma-Aldrich), and images were captured with a ChemiDocTM XRS+ System (Bio-Rad). All immunoblots were stripped with stripping buffer containing 62.5-mM Tris-HCL, 100-mM 2-mercaptoethanol, and 2% SDS (*w*:*v*, pH 6.7) at 50 °C for 30–45 min, and incubated with antibodies against β-actin or β-tubulin as loading controls. Three independent experiments were performed, each in triplicate with tissues prepared from different animals [30].

To obtain quantitative results the bands (representing each data point) were densitometrically scanned using the public domain ImageJ software (National Institutes of Health, Bethesda, MD, USA) [34]. The data obtained for each protein were normalized against its corresponding actin and expressed as relative intensity. The results of 10 separate measurements were expressed as mean ± SD.

### 2.5. Immunofluorescence

Immunofluorescence analysis was performed on MA-10 cells seeded on coverslips and treated as described in Section 2.3. The cells were washed with phosphate buffered saline (PBS) and fixed with methanol and acetone, as described previously [48,49]. Non-specific binding sites were blocked with 10% normal horse serum for 20 min at room temperature. Thereafter, to localize aromatase (P450arom), protein cells were incubated overnight at 4 °C with a primary antibody diluted in PBS (Table 1). On the next day, Alexa Fluor 488 goat anti-mouse IgG (1:200; Invitrogen, Waltham, MA, USA) was applied for 60 min. Lastly, coverslips were mounted with Vectashield mounting medium (Vector Labs.) with 4′,6-diamidino-2-phenylindole (DAPI) and examined with a Nikon epiflourescence microscope Eclipse Ni (Nikon Instech Co., Ltd., Tokyo, Japan). For negative control, primary antibody was omitted, and no fluorescence was observed (not shown).

### 2.6. Statistical Analysis

For normality measure, each variable was tested by using a Shapiro-Wilk W-test. Homogeneity of variance was assessed with Levene’s test. Since the distribution of the variables was not normal and the values were not homogenous in variance, all statistical analyses were performed using non-parametrical Kruskal-Wallis’s post-hoc comparison test to determine which values differed significantly from controls. The analysis was made using Statistica12 Software (Stat-Soft Inc., Tulsa, OK, USA). Data were presented as means ± SD. Data were considered statistically significant at *p* < 0.05. All the experimental measurements were performed in triplicates.

## 3. Results

### 3.1. Effect of GPER Agonist, GPER and PPAR Antagonists, and Estradiol on the Expression of GPER, PPARα and PPARγ in Mouse Testis Explants

**Figure 1:**
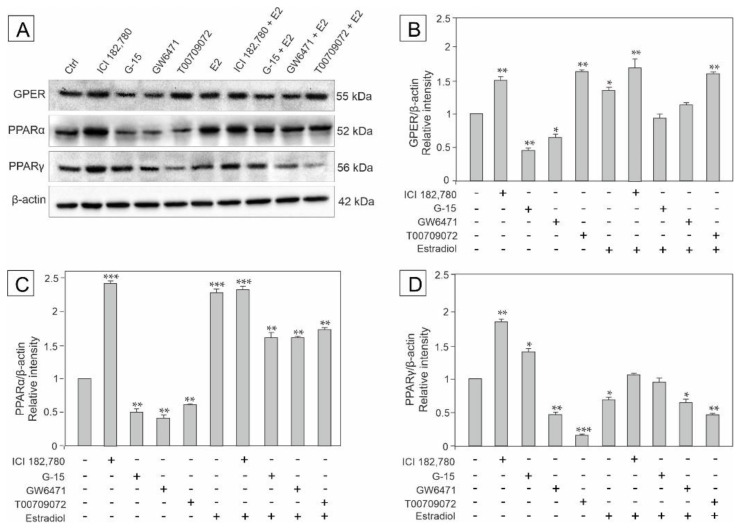
The effect of GPER agonist (ICI 182,780), GPER antagonist (G-15), PPARα antagonist (GW6471), PPARγ antagonist (T00709072), and estradiol (E2) on GPER, PPARα, and PPARγ protein expression levels in mouse testis explants (**A**–**D**). Representative blots of Western analysis (**A**) and relative expression of the proteins (**B**–**D**). Explants were harvested after 24 h. Untreated explants served as a control. Protein levels within control testis explants were given a value of 1. The cropped blots are displayed and the original blots are provided in Supplementary Materials (Figure S1). The relative levels of the proteins were normalized to β-actin which served as the internal protein loading control. The histograms are the quantitative representation of data (mean ± SD) of three independent experiments, each in triplicate (**B**–**D**). A plus sign (+) indicates the presence of the compound in the culture medium, a minus sign (–) indicates no compound in the culture medium. Asterisks indicate significant differences from control explants. Values are denoted as * *p* < 0.05, ** *p* < 0.01 and *** *p* < 0.001.

To determine receptors modulated by the compounds of agonistic and antagonistic properties and estradiol, the explants were incubated with GPER agonist (ICI 182,780), GPER antagonist (G-15), PPARα antagonist (GW6471), PPARγ antagonist (T00709072), and estradiol either alone or in combinations with each of the compounds (E2+ICI 182,780, E2+G-15, E2+GW6471, and E2+T00709072) as presented in Figure 1A–D. Of note, the compounds used are referred to hereafter as ICI, G-15, GW, and T00, respectively. As detected by western blot, immunodetectable proteins were observed as bands near 55 kDa (GPER), 52 kDa (PPARα), 56 kDa (PPARγ), and 42 kDa (β-actin) in the control and experimental groups of testis explants (Figure 1A; see respective bands). Protein band intensities were either markedly reduced or increased after treatment as determined quantitatively.

Densitometric analysis of protein content revealed significant decrease in GPER expression in the explants incubated with G-15 and GW added alone, compared to the control (*p* < 0.01, *p* < 0.05, respectively). In contrast, increased GPER expression was noted after treatment with ICI (*p* < 0.01), T00 (*p* < 0.01), estradiol alone (*p* < 0.05), and estradiol in mixture with either ICI or T00 (*p* < 0.01) compared to the control. On the other hand, the expression of GPER did not change when the explants were incubated in the presence of estradiol + G-15, and estradiol + T00 compared to the control (Figure 1B).

In turn, the expression of PPARα significantly decreased after treatment with G-15, GW, and T00 (*p* < 0.01) added alone, whereas the other treatments caused a marked increase (*p* < 0.001, *p* < 0.01) in the protein level when compared to the control (Figure 1C).

PPARγ expression increased in the explants incubated with ICI alone (*p* < 0.01) and in the presence of G-15 (*p* < 0.05), whereas markedly decreased expression was noted after treatments with GW (*p* < 0.01), T00 (*p* < 0.001), and estradiol in mixture with both, GW (*p* < 0.05) and T00 (*p* < 0.01), compared to the control. Decreased PPARγ expression was also observed when the explants were treated with estradiol alone (*p* < 0.05), however estradiol + G-15 and estradiol + ICI (*p* < 0.05) were ineffective in modulating this protein level (Figure 1D).

Based on these findings the expression of both receptor types (GPER and PPARα/PPARγ) seems to be directly modulated by estradiol and the receptor agonist and antagonists in various combinations (E2+ICI, E2+G-15, E2+GW, and E2+T00).

### 3.2. Effect of GPER Agonist, GPER and PPAR Antagonists, and Estradiol on the Expression of Raf, ERK1/2, PI3Kp85, and Akt in Mouse Testis Explants

**Figure 2:**
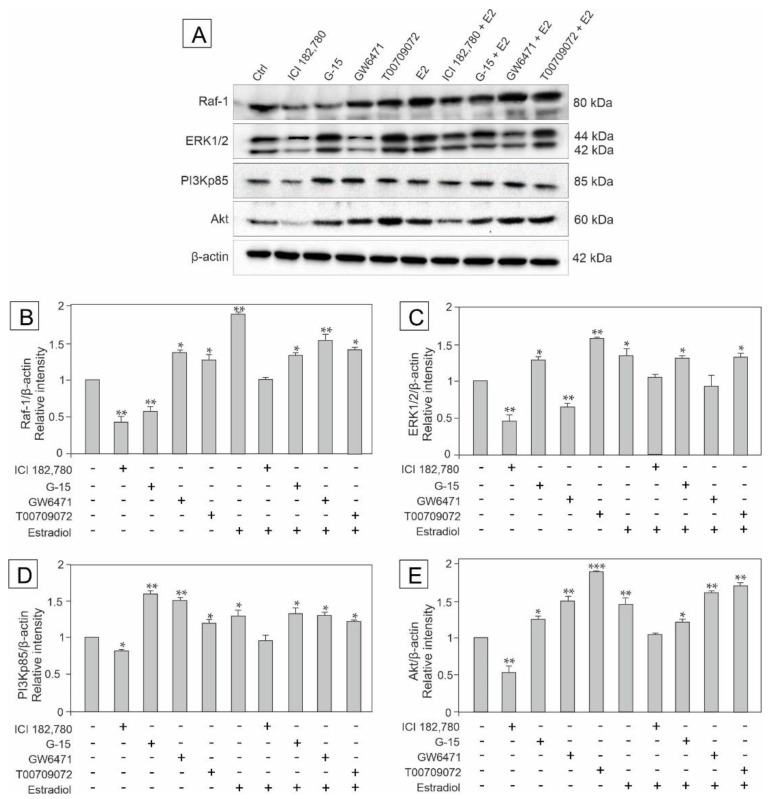
The effect of GPER agonist (ICI 182,780), GPER antagonist (G-15), PPARα antagonist (GW6471), PPARγ antagonist (T00709072) and estradiol (E2) on Raf-1, ERK 1/2, PI3Kp85, and Akt protein expression in mouse testis explants (**A**–**E**). Representative blots of Western analysis (**A**) and relative expression of the proteins (**B**–**E**). Explants were harvested after 24 h. Untreated explants served as a control. Protein levels within control testis explants were given a value of 1. The cropped blots are displayed and the original blots are provided in Supplementary Materials (Figure S1). The relative levels of the proteins were normalized to β-actin which served as internal protein loading control. The histograms are the quantitative representation of data (mean ± SD) of three independent experiments, each in triplicate (**B**–**E**). A plus sign (+) indicates the presence of the compound in the culture medium, a minus sign (–) indicates no compound in the culture medium. Asterisks indicate significant differences from control explants. Values are denoted as * *p* < 0.05, ** *p* < 0.01 and *** *p* < 0.001.

To determine signaling pathways engaged in the regulation of GPER and PPARs functions and to check whether estradiol has an impact on this regulation, the expression of kinases: Raf-1, ERK1/2, PI3Kp85, and Akt was analyzed in testis explants treated with ICI, G-15, GW, T00, and estradiol either alone or in combinations with each of the compounds (E2+ICI, E2+G-15, E2+GW, and E2+T00) as presented in Figure 2A–D.

Western blot analysis confirmed the presence of immunodetectable proteins of predicted molecular mass in control and experimental groups of the explants (Figure 2A; see respective bands). Quantitative analysis revealed a decrease in the expression of Raf-1 in the explants treated with G-15 or ICI alone (*p* < 0.01), however in the presence of estradiol + ICI, no changes in the protein level were found (Figure 2B). In contrast, the other treatments caused significant increase in the Raf expression (*p* < 0.05, *p* < 0.01) compared to the control. For details see Figure 2B.

In turn, the expression of ERK1/2 was significantly up-regulated in explants treated with G-15 (*p* < 0.05), T00 (*p* < 0.01), and estradiol either alone or in mixtures with G-15 and T00 (*p* < 0.05). Treatments with ICI and GW alone led to ERK1/2 down-regulation (*p* < 0.01). Neither estradiol in mixture with ICI nor with GW changed ERK1/2 expression compared to the control (Figure 2C).

As to the expression of PI3Kp85 and Akt, their protein levels decreased in explants treated with ICI (*p* < 0.05, *p* < 0.01, respectively), ICI + estradiol evoked no changes in both kinases expression, whereas the other treatments (E2+G-15, E2+GW, and E2+T00) led to the increase in PI3Kp85 and Akt expressions (*p* < 0.05, *p* < 0.01, *p* < 0.001) compared to the control (for details see Figure 2D–E).

These results indicate the involvement of the PI3K/Akt and MAPK pathway in the estradiol-mediated regulation of GPER and PPARs’ functions in mouse testis.

### 3.3. Effect of GPER Agonist, GPER and PPAR Antagonists, and Estradiol on the Expression of TGF-β, HIF-1α, VEGF and COX-2 in Mouse Testis Explants

**Figure 3:**
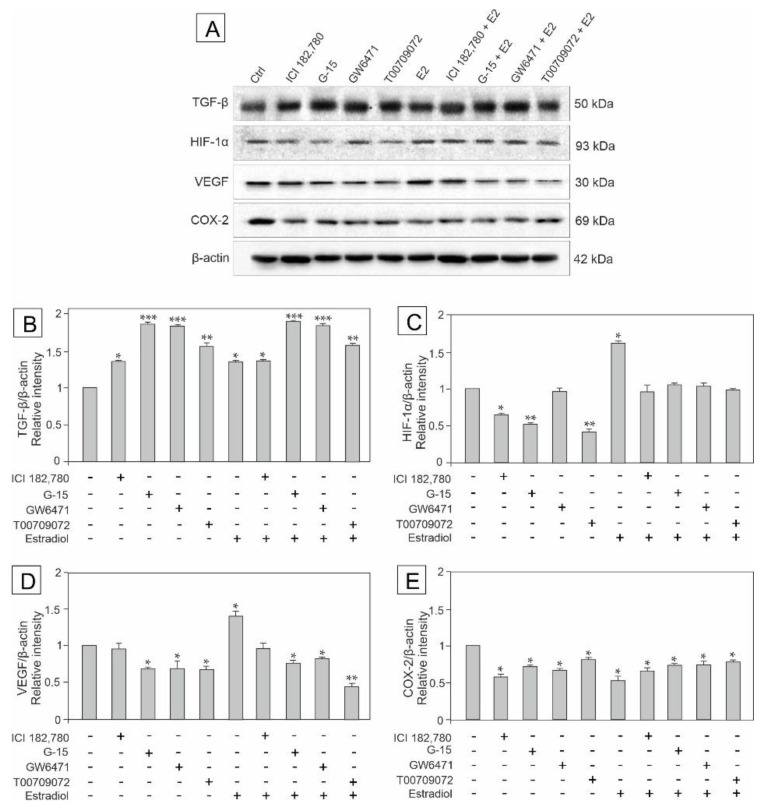
The effect of GPER agonist (ICI 182,780), GPER antagonist (G-15), PPARα antagonist (GW6471), PPARγ antagonist (T00709072), and estradiol (E2) on TGF-β, HIF-1α, VEGF and COX-2 protein expression in mouse testis explants (**A**–**E**). Representative blots of Western analysis (**A**) and relative expression of the proteins (**B**–**E**). Explants were harvested after 24 h. Untreated explants served as a control. Untreated explants served as a control. Protein levels within control testis explants were given a value of 1. Displayed are the cropped blots and original blots are provided in Supplementary Materials Figure S1. The relative levels of the proteins were normalized to β-actin which served as the internal protein loading control. The histograms are the quantitative representation of data (mean ± SD) of three independent experiments, each in triplicate (**B**–**E**). A plus sign (+) indicates the presence of the compound in the culture medium, a minus sign (–) indicates no compound in the culture medium. Asterisks indicate significant differences from control explants. Values are denoted as * *p* < 0.05, ** *p* < 0.01 and *** *p* < 0.001.

To determine whether GPER and/or PPAR signaling pathways are engaged in the regulation of potential inflammatory and/or carcinogenesis processes in mouse testis, and to check whether estradiol has an impact on this regulation, the expression of TGF-β, HIF-1α, VEGF and COX-2 was analyzed in the explants treated with ICI, G-15, GW, T00, and estradiol either alone or in combinations with each of the compounds (E2+ICI, E2+G-15, E2+GW, and E2+T00), as presented in Figure 3A–D.

Western blot analysis revealed the expression of TGF-β, HIF-1α, VEGF, and COX-2 in the tissue explants (Figure 3A), while densitometric analysis revealed quantitative changes in the protein levels as shown in Figure 3B–E. Interestingly, TGF-β expression was significantly up-regulated (*p* < 0.05, *p* < 0.01, *p* < 0.001), while the COX-2 expression was down-regulated (*p* < 0.05) in all experimental groups compared to the control (Figure 3B,E).

On the other hand, the increased expression of HIF-1α was observed only when estradiol was added alone (*p* < 0.05), while estradiol added as a mixture with each of the compounds (E2+ICI, E2+G-15, E2+GW, and E2+T00) caused no changes in the protein content, as did GW treatment. In contrast, incubations of explants with ICI (*p* < 0.05), G-15 (*p* < 0.01) or T00 (*p* < 0.01) led to the decrease in the HIF-1α protein levels compared to the control (Figure 3C).

VEGF overexpression (*p* < 0.05) was observed after treatment with estradiol alone (as it was in case of the HIF-1α) whereas ICI alone was ineffective in modulating the VEGF protein level. On the other hand, the expression of VEGF was significantly down-regulated after treatments with G-15 (*p* < 0.05), GW (*p* < 0.05), and T00 (*p* < 0.01) alone and together with estradiol (*p* < 0.05, *p* < 0.01) compared to the control (Figure 3D).

Altered expression of TGF-β, HIF-1α, VEGF, and COX-2 following estradiol administration, alone or in combination with the compounds of agonistic and antagonistic properties such as E2+ICI, E2+G-15, E2+GW, and E2+T00 (that disrupted the estrogen pathway) may suggest the induction of inflammatory and tumor formation processes in mouse testis.

### 3.4. Effect of Estradiol, GPER Agonist, and GPER and PPAR Antagonists on the Expression of GPER, PPARα and PPARγ in MA-10 Cells

**Figure 4:**
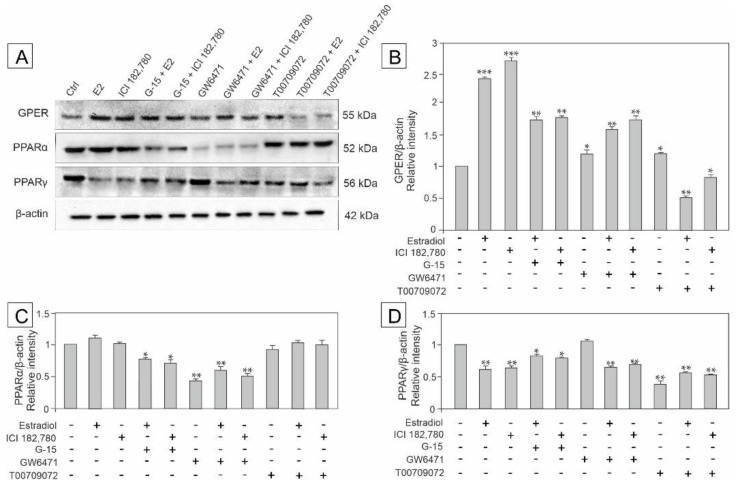
The effect of estradiol (E2), GPER agonist (ICI 182,780), GPER antagonist (G-15), PPARα antagonist (GW6471), and PPARγ antagonist (T00709072) on GPER, PPARα and PPARγ protein expression in MA-10 cells (**A**–**D**). Representative blots of Western analysis (**A**) and relative expression of the proteins (**B**–**D**). Cells were harvested after 24 h. Untreated cells served as a control. Protein levels within control cells were given a value of 1. The cropped blots are displayed and the original blots are provided in Supplementary Materials (Figure S1). The relative levels of the proteins were normalized to β-actin, which served as the internal protein loading control. The histograms are the quantitative representation of data (mean ± SD) of three independent experiments, each in triplicate (**B**–**D**). A plus sign (+) indicates the presence of the compound in the culture medium, a minus sign (–) indicates no compound in the culture medium. Asterisks indicate significant differences from control cells. Values are denoted as * *p* < 0.05, ** *p* < 0.01 and *** *p* < 0.001.

To determine changes in the expression of GPER and PPAR modulated by estradiol and the receptor agonist and antagonists, MA-10 cells were incubated with estradiol, ICI, G-15, GW, and T00 alone or in combinations with either estradiol or ICI (E2+G-15, E2+GW, E2+T00 or ICI+G15, ICI+GW, ICI+T00) as shown in Figure 4A–D. Immunodetectable proteins were detected by western blot (Figure 4A), while changes in band intensities were measured quantitatively (Figure 4B–D).

First, up-regulation of GPER expression was observed after almost all treatments and the changes were statistically significant (*p* < 0.05, *p* < 0.01, *p* < 0.001) in comparison with the control. Only the administration of estradiol in mixtures with either ICI or T00 led to GPER down-regulation (*p* < 0.01, *p* < 0.05, respectively) (for details see Figure 4B).

Second, a marked decrease in PPARα expression was noted after treatments with either GW alone or in combinations with estradiol and ICI (*p* < 0.01), as well as after combined treatments with G-15 + estradiol and G-15 + ICI (*p* < 0.05) when compared to the control. No significant changes in the expression of PPARα were observed in the remaining experimental groups (Figure 4C).

Third, the expression of PPARγ significantly decreased in almost all experimental groups (*p* < 0.05, *p* < 0.01) except for treatment with GW only, which did not affect the ex-pression of PPARγ (Figure 4D).

These results indicate that estradiol alone and in mixtures with GPER and PPARα/PPARγ ligands influences the expression of both receptor types (GPER and PPARs) affecting Leydig cell function.

### 3.5. Effect of Estradiol, GPER Agonist, GPER and PPAR Antagonists on the Expression of Raf-1, ERK1/2, PI3Kp85, and Akt Signaling Kinases in MA-10 Cells

**Figure 5:**
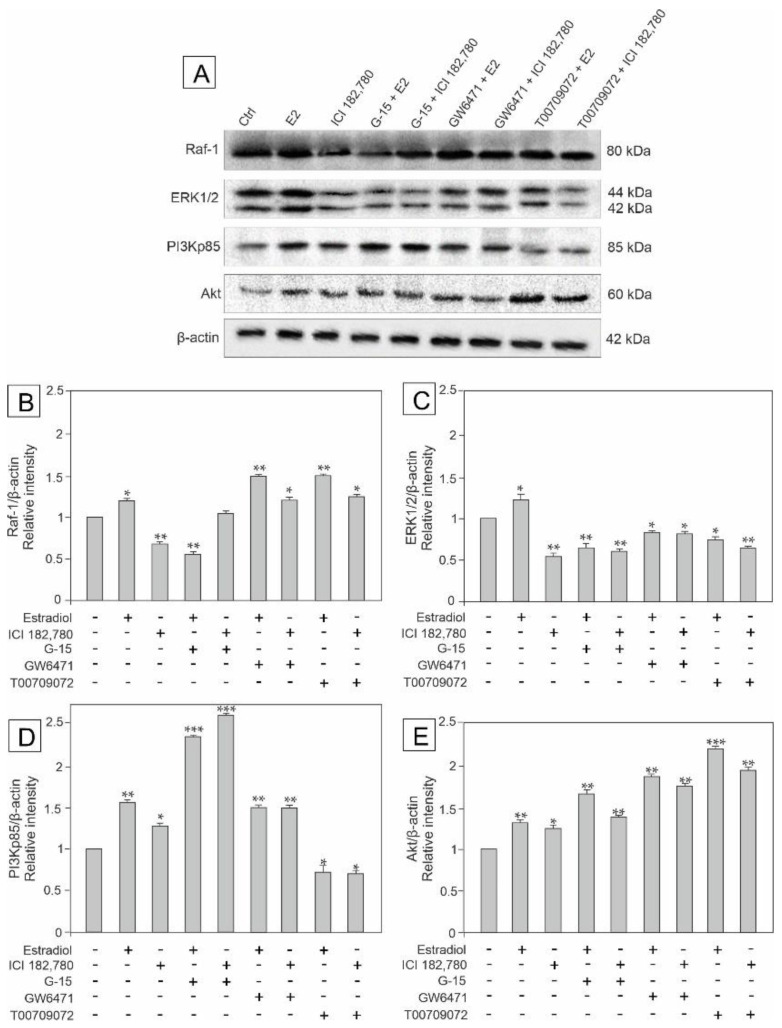
The effect of estradiol (E2), GPER agonist (ICI 182,780), GPER antagonist (G-15), PPARα antagonist (GW6471), and PPARγ antagonist (T00709072) on Raf-1, ERK 1/2, PI3Kp85, and Akt protein expression in MA-10 cells (**A**–**E**). Representative blots of Western analysis (**A**) and relative expression of the proteins (**B**–**E**). Cells were harvested after 24 h. Untreated cells served as a control. Protein levels within control cells were given a value of 1. The cropped blots are displayed and the original blots are provided in Supplementary Materials (Figure S1). The relative levels of the proteins were normalized to β-actin which served as internal protein loading control. The histograms are the quantitative representation of data (mean ± SD) of three independent experiments, each in triplicate (**B**–**E**). A plus sign (+) indicates the presence of the compound in the culture medium, a minus sign (–) indicates no compound in the culture medium. Asterisks indicate significant differences from control cells. Values are denoted as * *p* < 0.05, ** *p* < 0.01 and *** *p* < 0.001.

To determine signaling pathways engaged in the regulation of GPER and PPARs functions in MA-10 cells, and to check whether estradiol has an impact on this regulation, the expression of kinases: Raf-1, ERK1/2, PI3Kp85, and Akt kinases was analyzed in the cells incubated with estradiol, ICI, G-15, GW, and T00 alone and in combinations with either estradiol or ICI (E2+G-15, E2+GW, E2+T00 or ICI+G15, ICI+GW, ICI+T00) as shown in Figure 5A–E. Changes in the protein levels in MA-10 cells detected by western blots (as shown in Figure 5A) were confirmed quantitatively (Figure 5B–E).

The expression of Raf-1 was up-regulated after treatment with estradiol alone (*p* < 0.01) and in mixtures with either GW (*p* < 0.01) or T00 (*p* < 0.01), while estradiol in mixture with G-15 as well as ICI alone led to Raf-1 down-regulation (*p* < 0.01) compared to the control. When ICI was added in mixtures with the other compounds, the Raf-1 expression was either up-regulated (ICI+GW and ICI+T00) (*p* < 0.05) or unchanged (ICI+G-15) (Figure 5B).

In turn, the expression of ERK1/2 increased (*p* < 0.05) in estradiol-treated cells, and significantly decreased (*p* < 0.05; *p* < 0.01) in remaining groups compared to the control (Figure 5C).

Similarly to Raf-1 and ERK1/2 expressions, estradiol added alone led to significant up-regulation of PI3K expression (*p* < 0.01) *versus* control. Also estradiol in mixtures with G-15 or GW, ICI alone and ICI in mixtures with G-15 or GW markedly up-regulated (*p* < 0.05, *p* < 0.01, *p* < 0.001) PI3Kp85 expression, whereas treatments with either estradiol or ICI in mixture with T00 led to PI3Kp85 down-regulation (*p* < 0.05) compared to the control (for details see Figure 5D).

The expression of Akt was elevated in all treated groups (*p* < 0.01), with the most significant up-regulation after treatment with estradiol in mixture with T00 (*p* < 0.001) compared to the control (Figure 5E).

These results indicate that the GPER and PPARs’ functions in MA-10 cells are affected by estradiol alone or in mixtures (E2+G-15, E2+GW, and E2+T00) via PI3K/Akt and the MAPK signaling pathway.

### 3.6. Effect of Estradiol, GPER Agonist, and GPER and PPAR Antagonists on the Expression of TGF-β and COX-2 in MA-10 Cells

**Figure 6:**
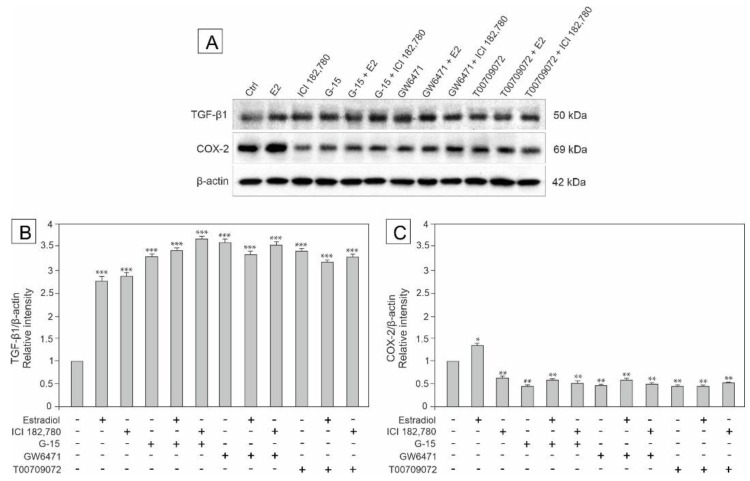
The effect of estradiol (E2), GPER agonist (ICI 182,780), GPER antagonist (G-15), PPARα antagonist (GW6471), and PPARγ antagonist (T00709072) on TGF- β and COX-2 protein expression in MA-10 cells (**A**–**C**). Representative blots of Western analysis (**A**) and relative expression of the proteins (**B**–**C**). Cells were harvested after 24 h. Untreated cells served as a control. Protein levels within control cells were given a value of 1. The cropped blots are displayed and the original blots are provided in Supplementary Materials (Figure S1). The relative levels of the proteins were normalized to β-actin which served as internal protein loading control. The histograms are the quantitative representation of data (mean ± SD) of three independent experiments, each in triplicate (**B**–**C**). A plus sign (+) indicates the presence of the compound in the culture medium, a minus sign (–) indicates no compound in the culture medium. Asterisks indicate significant differences from control cells. Values are denoted as * *p* < 0.05, ** *p* < 0.01 and *** *p* < 0.001.

To determine whether GPER and/or PPARs signaling pathways are engaged in the regulation of inflammatory and/or tumor formation processes in mouse MA-10 cells, and to check whether estradiol has an impact on this regulation, the expression of TGF-β and COX-2 was analyzed by western blot in cells incubated with estradiol, ICI, G-15, GW, and T00 alone and in combinations with either estradiol or ICI (E2+G-15, E2+GW, and E2+T00 or ICI+G15, ICI+GW, and ICI+T00 as shown in Figure 6A–C.

The expression of TGF-β was markedly up-regulated in all experimental groups (*p* < 0.001) (Figure 6B), while the COX-2 expression was only up-regulated in the estradiol-treated group (*p* < 0.05) and down-regulated in the remaining groups (*p* < 0.01) compared to the control (Figure 6C).

These results show a contribution of estradiol together with GPER and PPAR ligands to up-regulation of TGF-β expression and down-regulation of COX-2 expression, and suggest a potential role of these proteins in estradiol modulation of potential inflammatory and carcinogenic processes in mouse tumor Leydig cells.

### 3.7. Effect of Estradiol, GPER Agonist, and GPER and PPAR Antagonists on the Expression of Aromatase (P450arom) in MA-10 Cells

**Figure 7:**
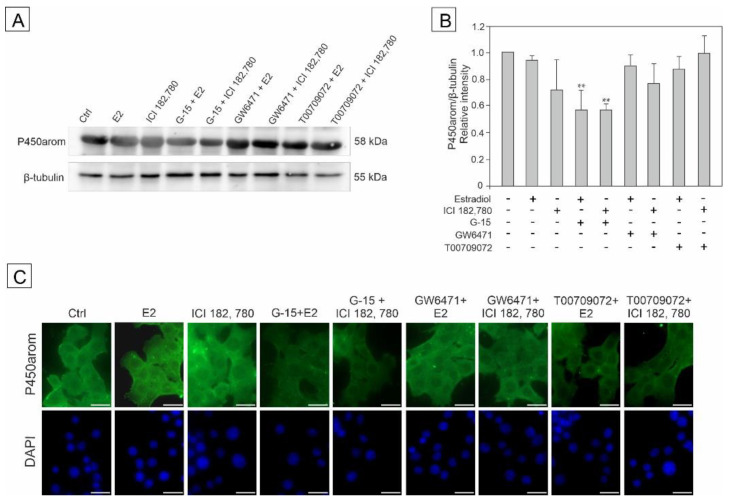
The effect of estradiol (E2), GPER agonist (ICI 182,780), GPER antagonist (G-15), PPARα antagonist (GW6471), and PPARγ antagonist (T00709072) on P450arom protein expression in MA-10 cells (**A**–**C**). Representative blots of Western analysis (**A**), relative expression of the protein (**B**), and immunofluorescent localization of the enzyme (**C**). Cells were harvested after 24 h. Untreated cells served as a control. Protein levels within control cells were given a value of 1. The cropped blots are displayed and the original blots are provided in Supplementary Materials (Figure S1). The relative levels of the proteins were normalized to β-tubulin, which served as the internal protein loading control. The histograms are the quantitative representation of data (mean ± SD) of three independent experiments, each in triplicate (**B**)**.** The scale bar = 20 µm (**C**)**.** A plus sign (+) indicates the presence of the compound in the culture medium, a minus sign (–) indicates no compound in the culture medium. Asterisks indicate significant differences from control cells. Values are denoted as ** *p* < 0.01.

To show the effect of estradiol and GPER/PPAR ligands of agonistic and antagonistic properties, the expression of aromatase (P450arom) in MA-10 cells was detected by western blot and immunofluorescence analyses. The cells were incubated with estradiol, ICI, G-15, GW, and T00 alone and in combinations with either estradiol or ICI (E2+G-15, E2+GW, and E2+T00 or ICI+G15, ICI+GW, and ICI+T00 as shown in Figure 7A–C.

The expression of P450arom was down-regulated in the cell groups incubated with G-15+E2 and G-15+ICI (*p* < 0.01) as confirmed quantitatively (Figure 7B), while in the remaining groups (*p* < 0.01) no statistically significant changes in P450arom immunoexpression were noted compared to the control (Figure 7B).

Immunofluorescence analysis revealed cytoplasmic localization of the enzyme both in control and treated cells. The intensity of the P450arom signal varied between groups and was less robust in G-15+E2, G-15+ICI, and T00+ICI-treated cells than that of the control (Figure 7C).

These results indicate that GPER antagonist (G-15) together with either estradiol or ICI has a potential to alter the expression of aromatase in MA-10 cells.

## 4. Discussion

Our study provides novel data about molecular mechanisms by which estradiol alone or together with GPER agonists and GPER and PPAR antagonists may modulate the expression of TGF-β, inflammation factor COX-2, and two factors implicated in vascularization and angiogenesis (VEGF and HIF-1α) via the MAPK and PI3K/Akt signaling in mouse testicular explants and mouse tumor Leydig cells.

It is well established that estradiol is a paracrine factor involved in the regulation of Leydig cell function. Pérez-Martínez et al. [50] postulated that an excess of estradiol in fetal mouse testis promotes Leydig cell hyperplasia. In transgenic mice with aromatase overexpression, Leydig cell hyperplasia was confirmed to be ERα mediated [51]. In the reported case, Maeda et al. [52] proposed elevated estradiol levels as likely tumor markers to detect the recurrence of human Leydig cell tumors (LCT). In a very recent study it was documented that almost 1/3 of patients with LCT had elevated estradiol levels [53]. In support, increased estradiol production (leading to signs of feminization) has also been reported in male dogs with Leydig cell tumors [54]. Recently, it has been demonstrated that estradiol signaling is also mediated by the estrogen receptor, GPER, in both normal and transformed somatic cells of the human testis [20], and, consequently, the role of GPER in the control of cell proliferation of testicular tissue has recently been established [55]. The authors concluded that GPER may be a potential target for the development of new pharmacological tools against testicular tumors. It is worth mentioning that we previously examined the relationship between estradiol level and GPER/PPAR expression in mouse testes with experimentally induced LCT following xenoestrogen bisphenol A and its derivatives treatment [30]. The altered expression of GPER/PPAR was related to increased estradiol production by the tumor tissue. There is also evidence that estradiol action via GPER leads to enhanced fibronectin matrix assembly in breast cancer cells and a high GPER expression is associated with the increased tumor recurrence [56,57]. It should be added that a GPER agonist—ICI 182,780 is well known as an estrogen antagonist which down-regulates classical ERs.

We previously demonstrated signaling pathway crosstalk between GPER and PPARs in mouse Leydig cells [29]. In the present study we showed that in testis explants, the expression of both, GPER and PPARα, is activated by the GPER agonist (ICI) and estradiol, while PPARγ expression is up-regulated by ICI, and down-regulated by estradiol. Our results also revealed the antagonistic effects of GPER, PPARα, and PPARγ blockers on the expression of GPER, PPARα, and PPARγ with exception of the PPARγ antagonist (T00), which acted agonistically (as the ICI did) on the GPER expression. We observed similar results in MA-10 cells, in which the effects of estradiol alone and ICI alone on the GPER expression were partially abolished by the GPER, PPARα, and PPARγ ligands. As revealed by Lappas and colleagues [58], PPARγ activation inhibits the release of the post-inflammatory cytokines interleukin-6 (IL-6), interleukin-8 (IL-8) and tumor necrosis factor-α (TNF-α) from tissues such as the amnion, placenta or chorion. Interestingly, Ma et al. [59] have demonstrated that in the ductal carcinoma, IL-6 and IL-8 serum levels were significantly higher (compared to the controls) and strongly correlated with the classical ER expression, clinical stage of tumor, and lymph node metastasis. It has also been reported that in human granulosa cells, PPARγ activation lowers the expression and activity of aromatase [60]. In the present study, treatment of testis explants with the PPARγ antagonist led to increased GPER and decreased PPARα expression levels. The level of PPARα was significantly lower after treatment with all the receptor antagonists (G-15, GW, and T00), however together with estradiol, the mixtures (E2+G-15, E2+GW, and E2+T00) were effective in increasing the PPARα expression levels. Similarly, it has been shown that activation of PPARα enhances cell proliferation in rat mammary glands and the human breast cancer cell lines [61,62]. It is worth noting that that a long-term exposure to PPARα-mediated herbicide (2,4-Dichlorophenoxy acetic acid) causes disruption of cholesterol/testosterone homeostasis in mouse Leydig cells and results in the development of Leydig cell tumors [63,64].

It is well established that TGF-β has a wide range of biological effects in regulating such processes as fertilization, as well as embryonic and organ development [65,66]. Related studies have shown that TGF-β1 is expressed in different testicular cells including Leydig cells, Sertoli cells, and germ cells [67]. Of note, TGF-β1 has also been reported to have multiple effects on the regulation of Leydig cell functions including inhibition of rat Leydig cell steroidogenesis in primary culture [68]. Moreover, the sustaining level of TGF-β in testis can be further influenced by gonadotropins and androgens, and may regulate testis function to maintain its homeostasis [69].

Our study showed that in mouse testis and MA-10 cells the expression of TGF-β was markedly elevated after treatments with ICI, estradiol and all the ligands used either alone or in combinations. High expression of TGF-β has also been demonstrated in metastatic primary prostate carcinoma compared to non-metastatic cancer [70]. Accumulating evidence suggests that the interplay of PPARs and TGF-β contributes to the regulation of cell proliferation, cell differentiation, and the associated cellular functions. For instance, the interaction of PPAR signaling with the proteins affected by up-regulated TGFβ receptor determines the outcome of the breast tumor progression [71]. Administration of PPARγ agonists (as therapeutic treatment in bronchopulmonary dysplasia) inhibits the canonical WNT/TGF-β pathway and stimulates PPARγ activity [72]. TGF-β1 as PPARγ, suppresses aromatase activity and the expression of the *Cyp19* gene as revealed in adult rat Leydig cells [73]. According to Gonzales et al. [74] TGF-β1 together with progesterone is involved in the hyperplasia and/or hypertrophy of Leydig cells via Smad1/5 signaling. In agreement, the high expression of TGF-β1 in patients with Leydig cell hyperplasia (LCH) and its correlation with estradiol-induced endoglin has been demonstrated, suggesting an involvement of TGF-β1 in the LCH [75]. Moreover, the abnormal expression of endoglin and its receptor complex has been shown as an early angiogenic switch indicator in premalignant lesions of the colon mucosa [76]. Interestingly, it has also been reported that the expression levels of TGF-β1 and its receptor as well as VEGF correlate with the malignant transformation of the uterine cervix [77].

In a further step of our study we demonstrated markedly decreased expression of COX-2 in all experimental groups of both mouse testis and MA-10 cells, except for the group of estradiol-treated cells in which the COX-2 expression increased. Different responses of ex vivo and in vitro models may result from the fact that in the ex vivo model several cell types are present. Although COX-2 expression level is the highest in the Leydig cells [78], the presence of this protein in Sertoli cells and germ cells was also detected in some studies [79,80]. Notably, both Sertoli cells and germ cells express estrogen receptors and PPARs, and therefore it is likely that agonist and antagonists of these receptors may also modify COX-2 expression in seminiferous tubules, which may contribute to the final outcome of our analyses.

It is known that COX-2 overexpression stimulates estrogen receptor expression in Leydig cell tumors (LCT), while inhibition of COX-2 down-regulates aromatase activity and lowers proliferation of LCTs [81]. It seems likely that decreased or unchanged P450arom expression in MA-10 cells obtained in the present study is related to diminished COX-2 expression and its potential to modulate the P450arom. Hermenegildo and co-workers [82] proposed that estradiol may increase COX-2 activity in the endothelium, leading to increased production of prostaglandin I2. The overexpression of COX-2 has also been shown in hormone-dependent tumors. It has been reported that COX-2 is overexpressed in endocrine-related primary prostate cancer with metastatic potential and may predict survival [70], while in human breast cancers, COX-2 overexpression is linked to VEGF overexpression and, therefore, tumor angiogenesis [83]. Accordingly, we demonstrated increased COX-2 and VEGF expression levels in the estradiol-treated groups and decreased expression of COX-2 and VEGF in the remaining experimental groups, indicating a correlation between COX-2 and VEGF expressions. It is tempting to speculate that alterations observed after estradiol treatment may be related to the induction of angiogenesis and tumorigenesis in the mouse testis. The ability of estrogenic GPER signaling to trigger HIF-1α-mediated VEGF expression has been reported as supporting angiogenesis and progression in breast cancer [27]. Kazi et al. [84] have recently postulated that rapid estradiol-mediated activation of PI3K/AKT signaling pathway and expression of HIF-1α protein induces VEGF expression in luminal epithelial cells of the rat uterus. Importantly, the activation of PPARγ expression may reduce the VEGF expression, thereby reducing angiogenesis. Both VEGF and its receptors regulate vasculogenesis, the development of blood vessels from precursor cells during early embryogenesis and angiogenesis, and both proteins are often overexpressed in tumors [85].

It is worth mentioning that GPER contributes to the angiogenic switch via mechanisms that include their functional interaction with HIF-1α and VEGF expressions [86]. Moreover, estrogenic signaling via GPER activates the HIF-1α/VEGF transduction pathway leading to angiogenesis and tumor growth [87]. Ten years ago functional cooperation between HIF-1α and GPER was demonstrated in cardiomyocytes and breast cancer cells. In the latter cells, GPER cooperates with HIF-1α to modulate VEGF in the hypoxic breast tumor microenvironment [17]. The simultaneous increase of the HIF-1α expression and elevated GPER and VEGF expressions was demonstrated in estradiol-treated endometrial cells [88]. It should be emphasized that these effects were abolished after GPER blockage. The above mentioned studies support the results of our experiment which revealed the up-regulation of GPER, VEGF and HIF-1α expression in mouse testis explants after estradiol treatments.

It is well established that selective estrogen receptor ligands can produce differential effects by acting on different receptors within one cell [89]. We previously demonstrated that in MA-10 cells with blocked GPER and PPAR activity, the PI3K-Akt-mTOR pathway was regulated diversely by different PPAR types, while progesterone secretion was always inhibited in these cells [29]. Next, we also showed that in experimentally-induced Leydig cell tumors (LCT) in mouse testis, estradiol may be controlled only via mTOR, similarly as in human LCT, however by different mechanisms [30]. Herein, we demonstrated up-regulation of the PI3K/Akt pathway in most of the experimental groups, except for the groups treated with either estradiol or ICI in mixtures with PPARγ antagonists, in which significant down-regulation in PI3K expression levels was noted in comparison to the control. These results indicate the relationship between estradiol and GPER/PPARs antagonist action. More precisely, G-15 acted antagonistically to estradiol in regulation of Raf-1 and PI3K expression, whereas GW and T00 displayed agonistic effects to estradiol. In turn, in the regulation of ERK1/2 and Akt expression, all the receptor antagonists acted agonistically to estradiol. One of the important biological consequences of GPER activation is the regulation of cell growth and apoptotic cell death [90]. In this process, estradiol binding to GPER causes G-protein complex dissociation, tyrosine kinase Src activation, and, finally, the activation of the MAPK pathway [91]. In turn, GPER activation induces the downstream signaling molecules such as MAPK and PI3K/Akt [92]. According to Yun et al. [93], activation of PKC, PI3K/Akt, and p44/42 MAPK pathways are required for induction of HIF-1α, leading to enhanced VEGF expression. Importantly, several different signaling pathways, e.g., PI3K/Akt and MAPK pathways, have been reported to be involved in VEGF gene activation via converging HIF-1α activation [94].

Collectively, this study, for the first time, has documented the effects of estradiol alone or in mixtures with GPER agonists (E2+ICI) and GPER/PPARs antagonists (E2+G-15, E2+GW, and E2+T00) on mouse testis and mouse MA-10 cells. The resulting up-regulation of TGF-β and down-regulation of COX-2 expressions may suggest impairment of aromatase activity and, in consequence, estrogenic imbalance in the testicular tissue and Leydig cells. Indeed, we have shown down-regulation of P450arom expression in MA-10 cells treated with GPER antagonists (G-15) together with either estradiol or ICI, and no changes after PPAR antagonists with either estradiol or ICI treatment which indicate GPER-mediated control of estradiol production in the cells. Our further results suggest that testis estradiol levels and their action via GPER, together with proper PPAR signaling, play a role in important physiological processes, being a key component in the maintenance of testis homeostasis and regulation of testis function. Moreover, changes in the expression of TGF-β and COX-2 suggest that disrupted estradiol-mediated pathway with simultaneously disturbed GPER-PPAR signaling after treatment with estradiol (either alone or in mixtures) may be related to tumorigenesis in the testis. It should be stressed that treatment with antagonists was aimed to disrupt physiological pathways of GPER and PPARs signaling in order to check whether these pathways could influence the expression of proteins involved in tumorigenesis.

The obtained results may set the direction for further research and indicate the aspects that should be taken into consideration while searching for factors involved in Leydig cell tumorigenesis in humans. Mouse models provide valuable information on the mechanisms related to the regulation of tumor development, but the extent to which these observations can be extrapolated to humans remains to be shown, and further studies using human LCT samples are required.

**Figure 8:**
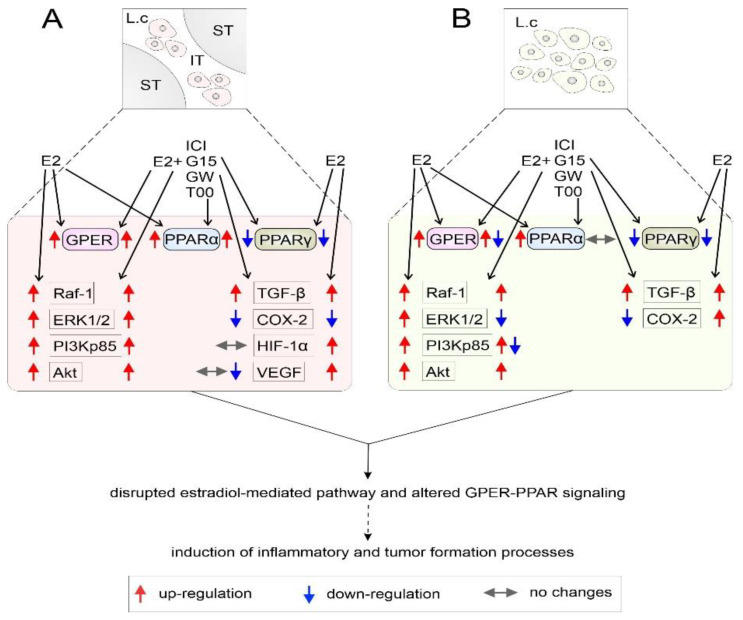
Schematic representation of local effects of estradiol alone or in combinations with GPER agonist and GPER and PPAR antagonists on the expression of Raf-1, ERK1/2, PI3Kp85 and Akt signaling proteins and TGF-β, COX-2, HIF-1α, and VEGF in mouse testicular tissue ex vivo (**A**) and mouse MA-10 Leydig cells in vitro (**B**)**.** Estradiol alone and in mixtures (E2+ICI, E2+G-15, E2+GW, and E2+T00) acting via GPER and PPARs induces the activation of Raf-1/ERK1/2 and PI3Kp85/Akt pathways and alters the expression levels of tumorigenic factors. The most likely effects on cellular processes are marked with a dotted arrow. GPER—G protein-coupled estrogen receptor; PPARα—peroxisome proliferator-activated receptor alpha; PPARγ—peroxisome proliferator-activated receptor gamma; ICI—GPER agonist; G-15—PPARα antagonist; GW—PPARα antagonist; T00—PPARγ antagonist; E2—estradiol; Raf-1—proto-oncogene serine-threonine-protein kinase; ERK1/2—extracellular signal-regulated protein kinase; PI3K—phosphatidylinositol 3-kinase; Akt—protein kinase B; TGF-β—tumor growth factor β; COX-2—cyclooxygenase-2; HIF-1α—hypoxia inducible factor 1-alpha; VEGF—vascular endothelial growth factor; ST—seminiferous tubule; IT—interstitial tissue; Lc—Leydig cells.

Finally, Figure 8 presents a schematic illustration of the local effects of estradiol alone or in combination with GPER agonist and GPER and PPAR antagonists on the expression of signaling proteins and tumorigenic factors in mouse testis explants and mouse MA-10 Leydig cells. We realize that the suggested effects are speculative and should be tested in a future study. However, disrupted estradiol-mediated pathways and altered GPER-PPAR signaling following estradiol treatment add new information about GPER-PPAR crosstalk within the testis.

## Figures and Tables

**Table 1:** Details for primary antibodies used for Western blotting and immunofluorescence.

Antibody	Host Species	Dilution	Vendor	Cat. No.
GPER	Rabbit	1:500 (WB)	Abcam (Cambridge, UK)	39742
PPARα	Mouse	1:500 (WB)	Thermo Fisher Scientific (Waltham, MA, USA)	MA1–822
PPARγ	Rabbit	1:500 (WB)	Abcam	209350
Raf-1	Rabbit	1:200 (WB)	Santa Cruz Biotechnology (Dallas, TX, USA)	sc-133
ERK1/2	Rabbit	1:500 (WB)	Cell Signaling Technology (Danvers, MA, USA)	9102
PI3Kp85	Rabbit	1:500 (WB)	Cell Signaling Technology	4292
Akt	Rabbit	1:500 (WB)	Cell Signaling Technology	9272S
TGF-β	Mouse	1:500 (WB)	Thermo Fisher Scientific	MA5-15065
COX-2	Rabbit	1:500 (WB)	Abcam	ab15191
VEGF	Rabbit	1:200 (WB)	Thermo Fisher Scientific	PA1-21796
HIF-1α	Mouse	1:500 (WB)	Thermo Fisher Scientific	PA1-16601
P450arom	Mouse	1:500 (WB) 1:50 (IF)	Bio Rad Labs	MCA2077S
β-actin	Mouse	1:3000 (WB)	Sigma–Aldrich	A2228
β-tubulin	Rabbit	1:500 (WB)	Sigma–Aldrich	T2200

GPER—G-coupled estrogen receptor, PPARα—peroxisome proliferator–activated receptor alpha, PPARγ—peroxisome proliferator–activated receptor gamma, Raf-1—RAF proto-oncogene serine/threonine-protein kinase 1, ERK1/2—mitogen-activated protein kinase, PI3Kp85—phosphatidylinositol-45-bisphosphate 3-kinase, Akt—Akt-serine/threonine-specific protein kinase (protein kinase B), TGF-β—transforming growth factor beta-1 proprotein, COX-2—cyclooxygenase-2, VEGF—vascular endothelial growth factor, HIF-1α—hypoxia-inducible factor 1-alpha, WB—Western blotting, IF—immunofluorescence.

## Data Availability

The data presented in this study are available on request from the corresponding author (E.G.-W.).

## Supplementary Materials

The following supporting information can be downloaded at: https://www.mdpi.com/article/10.3390/biomedicines10061390/s1, Figure S1: Uncropped images of western blots presented in Figure 1, Figure 2, Figure 3, Figure 4, Figure 5 and Figure 6. The cropped images shown in the main figures and their corresponding originals are versions taken at the same exposure level.
