# Supplementary material for: Modulatory Effects of Estradiol and Its Mixtures with Ligands of GPER and PPAR on MAPK and PI3K/Akt Signaling Pathways and Tumorigenic Factors in Mouse Testis Explants and Mouse Tumor Leydig Cells
Source: Biomedicines. 2022 Jun 12;10(6):1390. doi: 10.3390/biomedicines10061390 (PMC9219706; doi:10.3390/biomedicines10061390)
Supplement: Supplementary file 1 [file biomedicines-10-01390-s001.zip › biomedicines-1727405-supplementary.pdf]

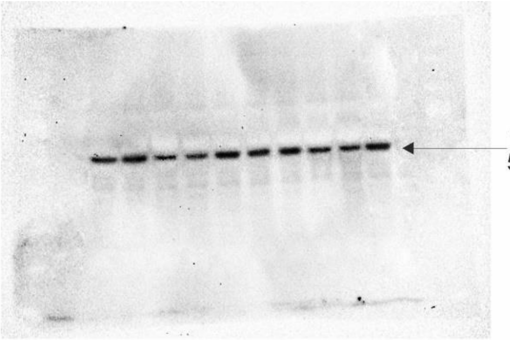
GPER
55 kDa

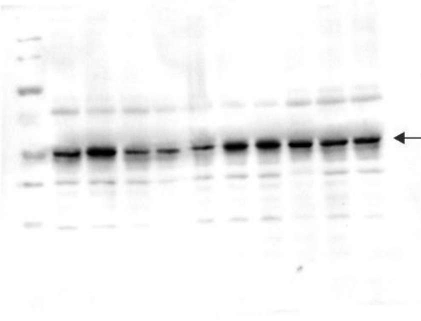
PPARα
52 kDa

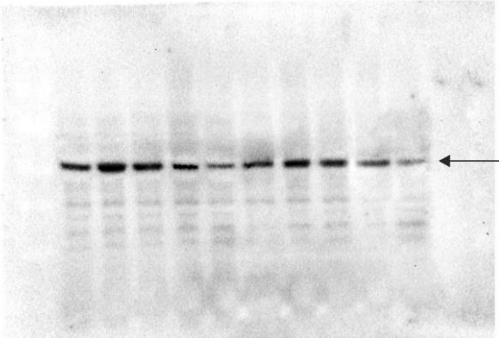
PPARγ
56 kDa

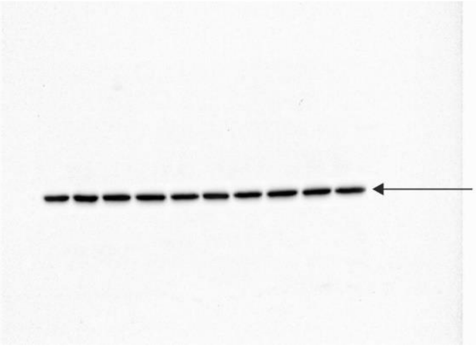
β-actin
42 kDa

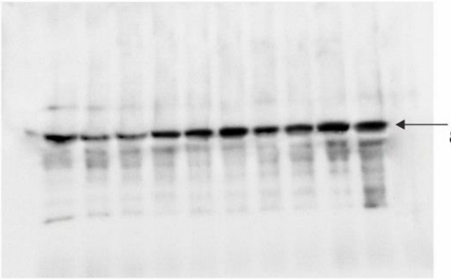
Raf-1
80 kDa

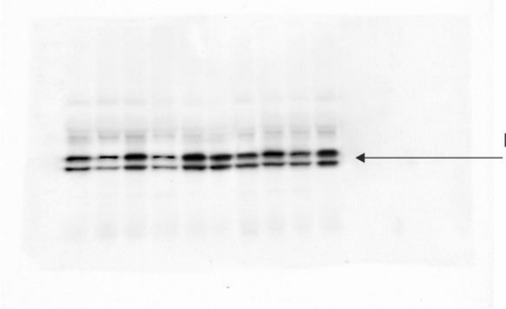
ERK 1/2
44 kDa
42 kDa

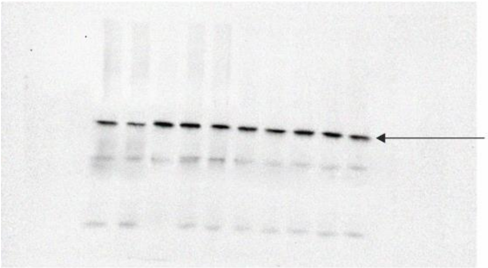
PI3Kp85
85 kDa

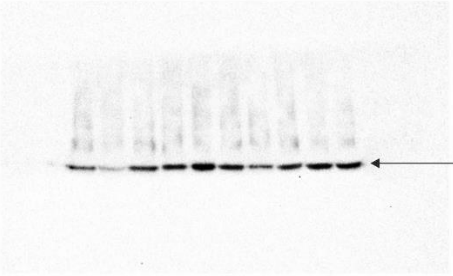
Akt
60 kDa

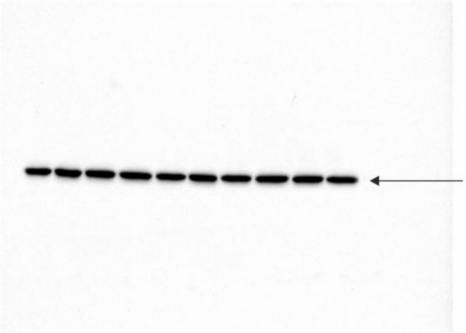
β-actin
42 kDa

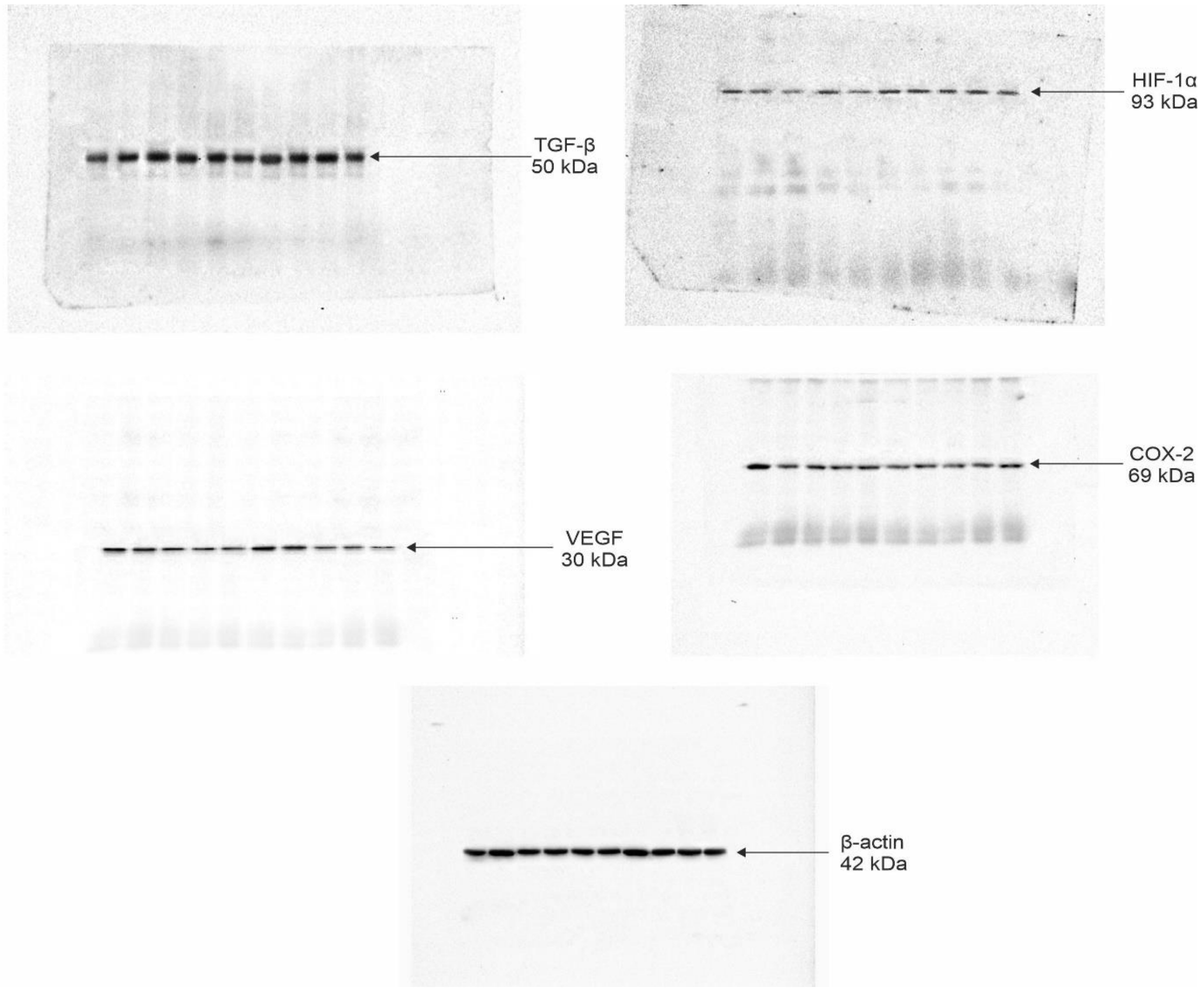
TGF-β
50 kDa
HIF-1α
93 kDa
VEGF
30 kDa
COX-2
69 kDa
β-actin
42 kDa

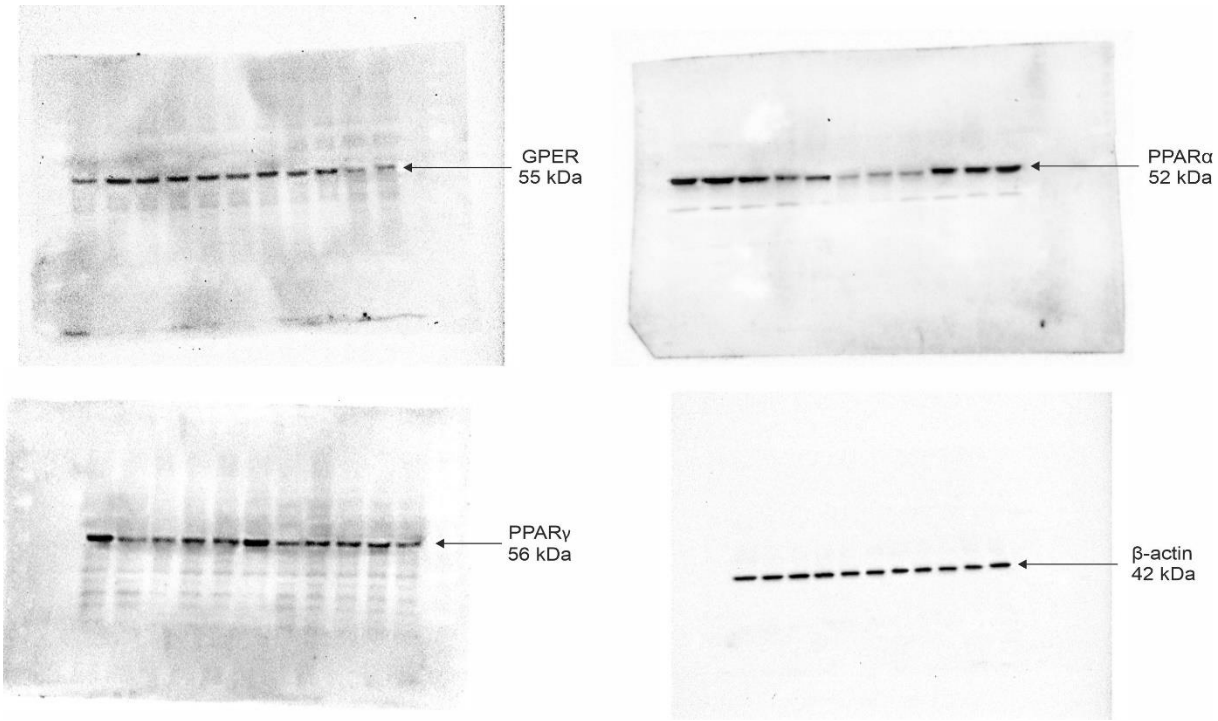
GPER
55 kDa
PPARα
52 kDa
PPARγ
56 kDa
β-actin
42 kDa

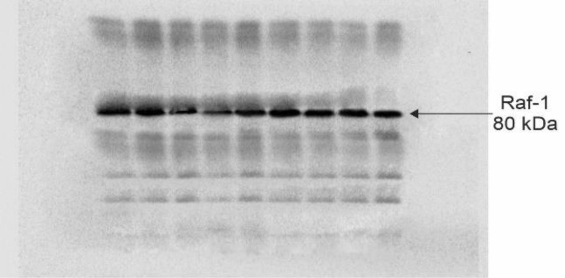
Raf-1
80 kDa

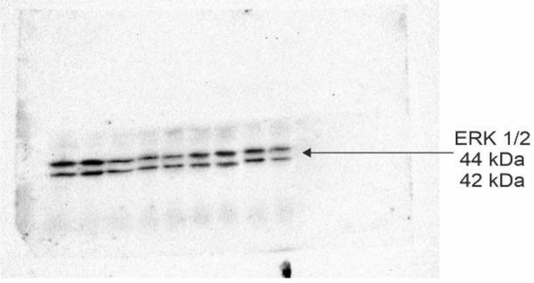
ERK 1/2
44 kDa
42 kDa

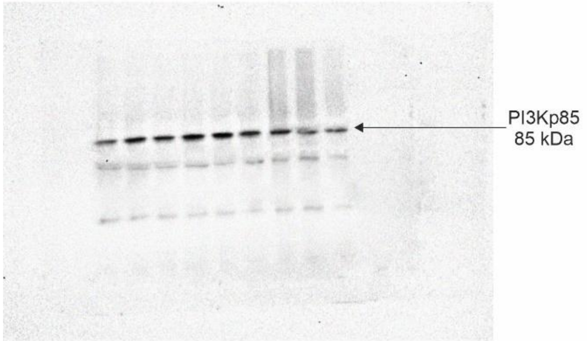
PI3Kp85
85 kDa

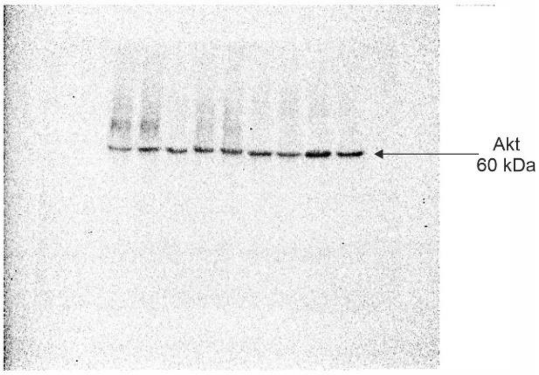
Akt
60 kDa

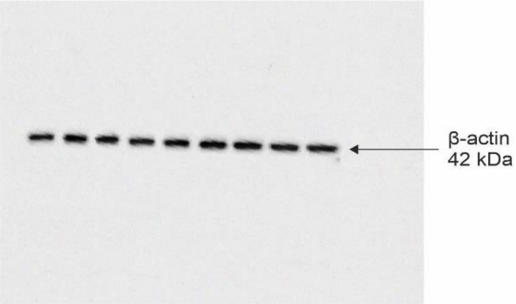
β-actin
42 kDa

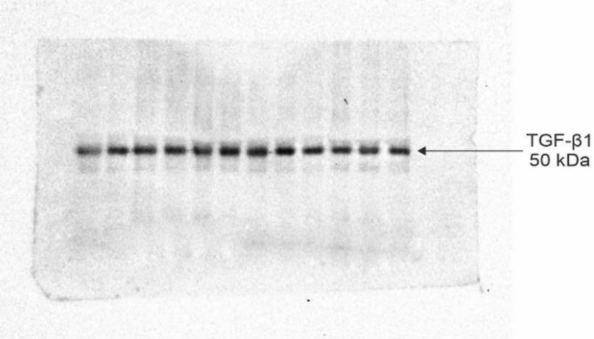
TGF-β1
50 kDa

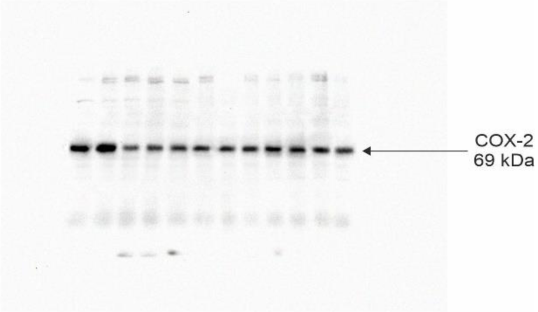
COX-2
69 kDa

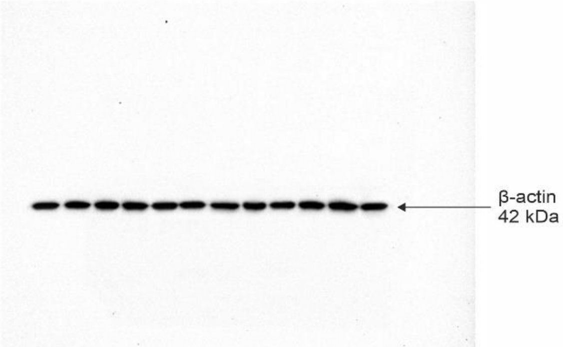
β-actin
42 kDa

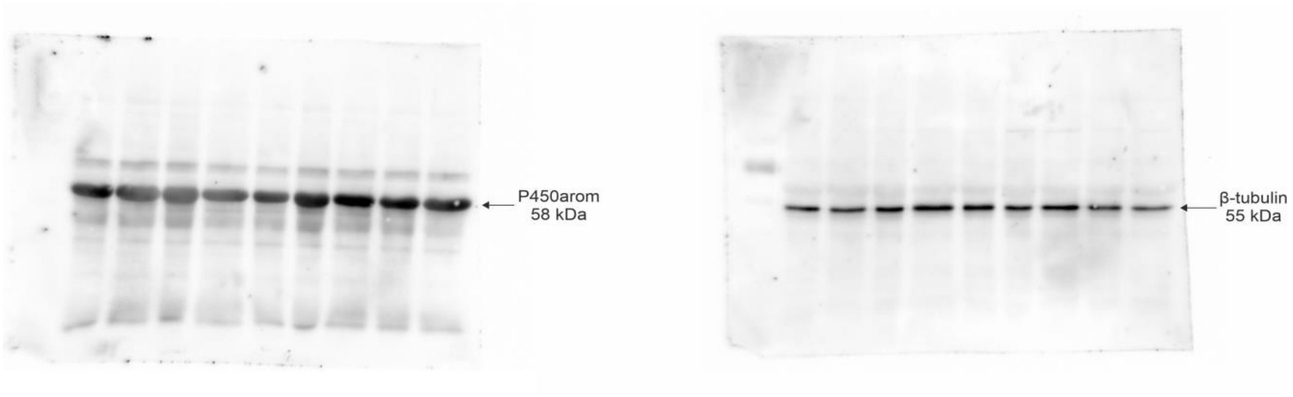

Figure S1. Uncropped images of western blots presented in Figures 1–6.
